# Fat mass: a novel digital biomarker for remote monitoring that may indicate risk for malnutrition and new complications in decompensated cirrhosis

**DOI:** 10.1186/s12911-023-02288-z

**Published:** 2023-09-13

**Authors:** K. Gananandan, V. Thomas, W. L. Woo, R. Boddu, R. Kumar, M. Raja, A. Balaji, K. Kazankov, R. P. Mookerjee

**Affiliations:** 1 Institute for Liver and Digestive Health, University College London, London, UK; 2 Royal Free Hospital, London, UK; 3 CyberLiver Limited, London, UK; 4 Department of Hepatology and Gastroenterology, Aarhus University Hospital, Aarhus, Denmark

**Keywords:** Remote monitoring, Body composition assessment, Decompensated cirrhosis

## Abstract

**Background:**

Cirrhosis is associated with sarcopaenia and fat wasting, which drive decompensation and mortality. Currently, nutritional status, through body composition assessment, is not routinely monitored in outpatients. Given the deleterious outcomes associated with poor nutrition in decompensated cirrhosis, there is a need for remotely monitoring this to optimise community care.

**Methods:**

A retrospective analysis was conducted on patients monitored remotely with digital sensors post hospital discharge, to assess outcomes and indicators of new cirrhosis complications. 15 patients had daily fat mass measurements as part of monitoring over a median 10 weeks, using a Withing’s bioimpedance scale. The Clinical Frailty Score (CFS) was used to assess frailty and several liver disease severity scores were assessed.

**Results:**

73.3% (11/15) patients were male with a median age of 63 (52–68). There was a trend towards more severe liver disease based on CLIF-Consortium Acute Decompensation (CLIF-C AD) scores in frail patients vs. those not frail (53 vs 46, *p* = 0.072). When the cohort was split into patients who gained fat mass over 8 weeks vs. those that lost fat mass, the baseline CLIF-C AD scores and WBC were significantly higher in those that lost fat (58 vs 48, *p *= 0.048 and 11.2 × 10^9^ vs 4.7 × 10^9^, *p* = 0.031).

**Conclusions:**

This proof-of-principle study shows feasibility for remote monitoring of fat mass and nutritional reserve in decompensated cirrhosis. Our results suggest fat mass is associated with greater severity of acute decompensation and may serve as an indicator of systemic inflammatory response. Further prospective studies are required to validate this digital biomarker.

## Background

Malnutrition has been estimated to be present in 20% of patients with compensated cirrhosis and between 60–90% of individuals with decompensated cirrhosis (defined by the development of ascites, hepatic encephalopathy (HE), gastrointestinal bleeding or bacterial infection) [1–3] This is due to reduced dietary intake secondary to anorexigenic effects of inflammatory cytokines and malabsorption secondary to impacts on the gut through portal hypertension, resulting in increased lipolysis of fat and proteolysis of skeletal muscle [4, 5]. These effects lead to fat wasting and sarcopenia, named protein-energy malnutrition (PEM). The group of particular concern are those with decompensated cirrhosis where median survival reduces from 10 to 2 years compared to individuals who are compensated, and re-admission rates following acute decompensation are as high as 30–50% [6–8].

PEM has been associated with increased mortality and as well as decompensation events in the form of ascites, gastrointestinal bleeding, and HE in patients with liver cirrhosis [9–11]. This effect is partly due to a loss of muscle mass which is termed sarcopaenia [12]. Sarcopaenia is a surrogate marker for severe malnutrition and a dominant component of frailty [13]. As well as muscle depletion, loss of fat mass is likely to be important, with evidence showing it may be protective against sarcopaenia as an alternative essential energy source [14]. Indeed, there is evidence to suggest that in earlier stages of cirrhosis it is predominantly fat wasting that occurs, which may drive the muscle depletion in more advanced stages of the disease [15].

Given the morbidity and mortality associated with malnutrition in liver cirrhosis, regular nutritional assessment is imperative. However, this is often resource heavy, time-consuming, and often difficult to prioritise in an overstretched healthcare system, especially in the outpatient setting. Dual-energy X-ray absorptiometry (DEXA) is the reference standard for nutritional assessment but is often unavailable, expensive and uses ionising radiation, whereas anthropometric measures often underestimate malnutrition in this population [16]. The utility of remote monitoring has not been evaluated in this area and is in keeping the NHS long term plan to digitise health care and deliver sustainable healthcare into the community [17].

Bioelectrical Impedance Analysis (BIA) is a quick, convenient, and cost-effective way of measuring body composition and has demonstrated accuracy in the cirrhotic population [16, 18] BIA provides fat mass (FM) and fat-free tissue mass (FFM) and works on the principle that electrical conductivity is reduced in fat tissue due to increased resistance (impedance), in contrast to FFM which has much more rapid conduction [19] Studies that have assessed BIA in liver cirrhosis have tended to do assessments at a single time point. This is the first study to assess whether regular monitoring of fat mass remotely in the community, could be used as a biomarker to assess patients with greater risk of new cirrhosis complications.

## Methods

A retrospective analysis was conducted on patients being remotely monitored at the Royal Free Hospital between August-December 2020, with CirrhoCare, in partnership with CyberLiver Limited [20]. 20 patients were monitored remotely for 12 weeks post hospital discharge for acute cirrhosis decompensation. This was the first ever study of digital, multi-modal monitoring, at home for management of advanced cirrhosis. It involved monitoring a range of vital signs (sensor-technology), fluid balance (bioimpedance-scale) and higher mental function (smartphone-app) in the patient's home, all key metrics perturbed in advanced cirrhosis. The data was uploaded in real-time securely to CyberLiver’s secure cloud and platform. The actionable, decision-assisted, analytical algorithms, then suggested interventions on a clinician dashboard (assessing clinical outcomes), for community-based therapy.

BIA data was obtained using a Withing’s bioimpedance scale. Participants were prompted via the CirrhoCare App to measure their weight and body composition at the same time every day, by standing in the centre of the scales in bare feet and minimal clothing. The scales provided measurements of total weight, fat mass, muscle mass, bone mass and hydration status. The average of all fat mass measurements taken during the 1^st^ week of the study for each participant was calculated to provide an average week 1 fat mass. The same was done for week 8 to calculate a week 8 fat mass. Food intake data was collected through self-reporting. Patients entered the number of cooked meals and number of total meals they had daily. Alcohol intake during the study period, if applicable, was also recorded.

The Clinical Frailty Score (CFS) was used to assess frailty with a score of 5 or more being used to define frailty, which is consistent with the literature [21, 22]. Sarcopaenia was assessed by the Skeletal Mass Index (SMI) from CT imaging either during admission or < 3 months prior to admission [23]. SMI has been validated and gives a robust measure of whole-body muscle mass by measuring the cross-sectional area of muscle at the level of the third lumbar vertebrate (L3), normalized to the patient’s height. Sarcopaenia was defined as per the American Association for the Study of Liver Diseases (AASLD) guidelines (SMI: men < 50cm^2^/m^2^, women < 39cm^2^/m^2^) [12].

Each participant had routine face-to face clinical assessments and blood tests performed at baseline and end of participation. Clinical examination was also undertaken including assessments of frailty and cirrhosis decompensation. Liver severity scores: Child–Pugh (CP), Model-For-End-Stage Liver Disease (MELD) and EF-CLIF Acute Decompensation score (CLIF-C AD*)* were then calculated at baseline and follow-up [2, 24, 25]. Information regarding any clinically significant events or hospital re-admissions during the trial period were also obtained.

Data analysis was performed using IBM SPSS software. A non-parametric assumption was used for all statistical tests. A Pearson’s chi-squared was used to test for statistically significant differences in nominal or ordinal data and the Mann–Whitney U test was used for continuous data. Spearman’s test was used to assess for correlations.

## Results

Fifteen patients were included for analysis with complete BIA data available for 8 weeks. Table 1 shows a summary of their baseline characteristics. Most patients were male (73.3%) with a median age of 63 (IQR 52–68). The underlying aetiology of liver cirrhosis in the majority was alcohol (80%). All patients had ascites to varying degrees at recruitment with only 1 individual diagnosed with refractory ascites. 6/15 (40%) patients had low-grade HE at baseline (grade 1), with all individuals assessed as having the capacity to participate in the monitoring program by the investigating team.

**Table 1:** Baseline characteristics of participants

Characteristic	Value
Age	63 (52–68) years
Male	11/15 (73.3%)
Alcohol aetiology of cirrhosis	12/15 (80%)
Frailty present	8/15 (53.3%)
CFS	5 (3–5)^a^
BMI	24.9 (22.7–26.6) kg/m^2a^
CP score	8 (8–9)^a^
MELD score	12 (10–19)^a^
CLIF-C AD score	49 (46–58)^a^
CRP	8 (1.8– 19.3) mg/L^a^
WBC	5.29 (3.6–10.5) x10^9a^
Urea	4.8 (2.7–6.4) mmol/L^a^
Creatinine	80 (60–99) umol/L^a^
Albumin	33 (30–35) g/L^a^
Week 1 Fat mass average	13.94 (10.6–17.1) kg^a^

^a^Median values provided with interquartile ranges (IQR)

### Bioimpedance data

BIA data provided total body weight values which were the sum of fat mass, bone mass, and muscle mass with hydration status determined separately. As muscle mass trends directly mirrored hydration, and muscle is the only hydrous element of the three body compartments, it was concluded that the muscle mass component also included total body water. The values for muscle mass were therefore deemed unreliable as liver cirrhosis patients often have significant fluid accumulation, and therefore this was not analysed. Bone mass trends were also found to be influenced by trends in hydration and so analysis of trends in this component was not undertaken. Although bone is an anhydrous body compartment, it is a very small mass in comparison to hydration, and as such, readings may have been affected by large fluid shifts which do occur in this population. By contrast, fat mass trends did not mirror hydration trends and were, therefore, used for analysis.

### Frailty

8/15 (53.3%) of patients were defined as frail. The median CFS score was 5 (IQR 5–5.5) in the frail group vs 3 (IQR 3–4) in the non-frail group. The body mass index (BMI) was similar across both groups (24.8 vs 25.1) with no significant differences in admission white blood cells (WBC), C-reactive protein (CRP), Albumin, Urea and Creatinine (Cr) noted. The admission CLIF-C AD showed a trend towards being higher in the frail cohort compared to the non-frail group (53 (IQR 48.5–59.0) vs 46 (IQR 43.7–51.2), *p* = 0.072). In addition, 3/15 patients had hospital re-admissions during the study period due to decompensation of cirrhosis and all these individuals were frail.

The average week 1 and week 8 fat masses were higher in the non-frail cohort in comparison to the frail cohort (17.1 vs 13.0 kg, 17.9 vs 14.6 kg respectively), although this was not statistically significant. There was no significant difference in change in fat mass over the study period for the two groups.

### Sarcopaenia

9/15 patients had CT imaging available for assessment of sarcopenia within the specified time frame. 6/9 (66.7%) patients had sarcopaenia based on the AASLD guidelines imaging criteria [12]. Baseline BMI was significantly lower in the sarcopaenic group vs non-sarcopaenic group (23 vs 28 kg/m^2^, *p* = 0.048). In addition, there was a trend towards a lower creatinine in the sarcopaenic group (74 vs 99 μmol/L, *p* = 0.09). Whilst median CLIF-C AD score was higher in the sarcopaenic cohort, this was not statistically significant (53 vs 47). There were no other significant differences in baseline blood results and hospital re-admission rates between the two groups.

The average week 1 and week 8 fat masses were lower in the sarcopaenic group (12.5 vs 17.1 kg, 13.8 vs 17.9 kg respectively). The sarcopaenic cohort gained 1.44 kg over the study period compared to 0.8 kg in the non-sarcopaenic group which corresponded with a higher number of cooked meals (2.5 vs 1) and total meals (3 vs 2) per day. However, none of these changes were significant.

### Fat mass changes

A comparative analysis was performed between those individuals that gained fat mass during the 8-week study period vs. those that lost fat mass (12 vs 3 participants). There was no significant difference between the number of meals and cooked meals per day between the two groups (2.5 vs 2 and 1.5 vs 2). In addition, no difference in frailty was noted across the two groups with the median CFS score being 5 in both groups. No significant correlations were noted between week 1, week 8 and change in fat mass in relation to admission blood test results, sex or liver disease severity scores.

**Fig. 1:**
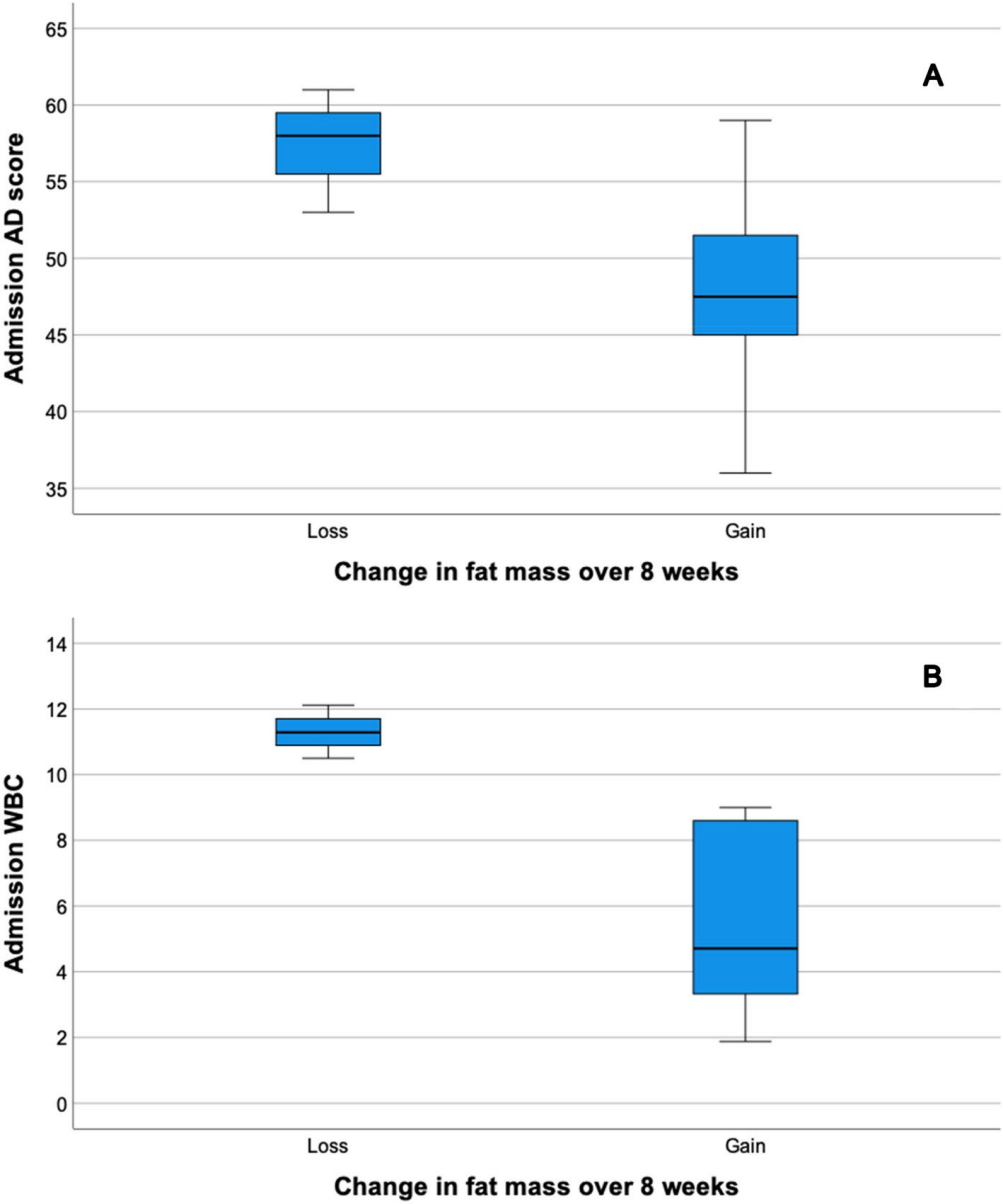
Graphs showing differences in admission CLIF-C AD scores (1A) and admission WBC (1B) in individuals who lost weight over 8 weeks versus those who gained weight

Admission CLIF-C AD score and WBC were significantly higher in individuals who lost fat compared to those who gained fat (58 vs 48, *p* = 0.048 and 11.2 vs 5.0, *p* = 0.031), which can be seen in Fig. 1.

## Discussion

This study demonstrates that BIA data can be obtained accurately and remotely in the decompensated cirrhosis population over a 3-month time period. BIA has previously been studied in the cirrhotic population and has demonstrated that it could play an important role in nutritional assessment as well as correlating with mortality [26–28]. However, these studies have predominantly assessed FFM (bone, muscle, and total body water) and only at a single time point. This pilot study is the first to demonstrate the potential benefit of monitoring fat mass in this population and shows that it can be monitored accurately in the patient’s home on a daily basis. Patients with decompensated cirrhosis frequently suffer from complications such as ascites and peripheral oedema, with significant fluid shifts following abdominal paracentesis. There is concern in the field about fluid retention affecting the accuracy of BIA results, although some studies have demonstrated it can be used in patients with ascites [26–29]. Indeed, in this study muscle and bone mass tended to mirror hydration status and, therefore, were not analysed as they were felt to be inaccurate. By comparison, fat mass was not affected by hydration status, and therefore would seem to be the ideal component of BIA analysis to use in the decompensated cirrhosis population.

Frailty is a broad syndrome which involves decreased physiological reserve, resulting in increased vulnerability to health stressors and predisposes to adverse health outcomes [30]. In cirrhosis, there has been a tendency to focus on physical frailty as opposed to the traditional care-of the elderly definition, which is more of a global construct [12]. 53.3% of patients is this study were defined as frail which is higher that the 17–43% reported in the literature [31, 32]. This is potentially because patients were recruited to this study following an admission with an acute decompensation of cirrhosis and, therefore, were likely to be deconditioned with more advanced disease. Indeed, all hospital re-admissions were in the frail cohort and there was a trend towards higher recruitment CLIF-AD scores in comparison to the non-frail individuals. This aligns with the literature which shows that frailty is associated with increased morbidity and cirrhosis complications [33, 34].

The proportion of sarcopaenic patients in the study is consistent with the literature which suggests an incidence of 30–70% [35]. Our results show that the sarcopaenic group had a significantly lower creatinine than the non-sarcopaenic group. This is not surprising given that skeletal muscle mass is the main determining factor of creatinine generation and therefore a low muscle mass would lead to reduced creatinine levels [36]. Lower week 1 and week 8 average fat masses were also noted in the sarcopaenic group, although non-significant, which is potentially due to the small sample size. This is logical given the link between protein and lipid metabolism. Cirrhosis is a condition with impaired responses to fasting and accelerated starvation responses. Sarcopaenia results in reduced metabolic reserves and in order to preserve muscle, adipose tissue is metabolised preferentially [37]. Indeed, there is evidence that preventing fat wasting may be protective against sarcopaenia [14]. The lower combined muscle and fat mass in the sarcopaenic cohort would explain why this cohort had a significantly lower BMI than the non-sarcopenic group. We did not demonstrate any significant difference in weight change and meal intake over the study period in the sarcopaenic vs non-sarcopaenic cohort. Although we are not able to prove this, we theorise that regular monitoring did have a positive impact on patient behaviour including diet this would need to be explored in further prospective studies.

This study demonstrated that both admission WBC and CLIF-C AD score were significantly higher in those individuals that lost fat mass over the study period compared to those who gained it. This is not necessarily surprising as it is known that decompensated cirrhosis is associated with systemic inflammation and this progresses with liver disease severity [3, 38]. It is also known that that this hyperinflammatory state is an energetically expensive process [39]. This higher level of catabolism with increasing liver disease severity is associated with increased mobilisation and oxidation of fat substrates and higher levels of PEM [40, 41]. It therefore follows that those with more severe liver disease are more likely to lose fat mass over time. Whilst this theory is established in the literature, previous studies have tended to assess fat mass at a single time point. This seems to be the first study to demonstrate this relationship in the decompensated cirrhosis population with regular fat mass monitoring in the community, and suggests this could be used as a biomarker, to assess disease stability or/and response to management.

The main limitation of this study is that it draws upon a small sample size, and over a 10–12 week study duration. It would have also been advantageous to have another formal nutritional assessment at baseline, such as anthropometry or DEXA scan for comparison, albeit these were not within the CirrhoCare study protocol, from which the data for analysis was derived. In addition, whilst there has been some concern about the accuracy of BIA in the cirrhosis population due to fluctuating hydration status, this has already been addressed earlier in the discussion. Whilst fluctuations in hydration seemed to alter muscle and bone mass, fat mass seemed to be unaffected in our population.

In conclusions, this pilot study shows feasibility for remotely monitoring of fat mass and nutritional reserve in decompensated cirrhosis. Our results suggest that fat mass is associated with the severity of acute decompensation and may serve as an indicator of systemic inflammatory response. BIA is ideal to monitor fat mass, as it is safe, rapid, and requires little to no training and can be repeated. Further prospective studies are required to validate this digital biomarker in identifying and helping prevent malnutrition in this vulnerable cirrhosis population with a high morbidity and mortality.

## Data Availability

The datasets used and/or analysed during the current study are available from the corresponding author on reasonable request.

## Abbreviations

AASLD: American Association for the Study of Liver Diseases
BIA: Bioelectrical Impedance Analysis
BMI: Body mass index
CFS: Clinical Frailty Score
CLIF-C AD: EF-CLIF Acute Decompensation score
CP: Child–Pugh
Cr: Creatinine
CRP: C-reactive protein
DEXA: Dual-energy X-ray absorptiometry
FF: Fat mass
FFM: Fat-free tissue mass
HE: Hepatic encephalopathy
L3: Third lumbar vertebrate
MELD: Model-For-End-Stage Liver Disease
PEM: Protein-energy malnutrition
SMI: Skeletal Mass Index
WBC: White blood cells
